# The Role of CCL21/CCR7 Chemokine Axis in Breast Cancer Progression

**DOI:** 10.3390/cancers12041036

**Published:** 2020-04-23

**Authors:** Balsam Rizeq, Mohammed Imad Malki

**Affiliations:** College of Medicine, QU Health, Qatar University, P. O. Box. 2713, Doha, Qatar; brizeq@qu.edu.qa

**Keywords:** CCR7, CCL19, CCL21, breast cancer, metastasis, signaling therapy

## Abstract

Breast cancer is a leading cause of cancer-related deaths worldwide, predominantly caused by metastasis. It is generally accepted that the pattern of breast cancer metastasis is largely determined by the interaction between the chemokine receptors on cancer cells and the chemokines expressed at the sites of metastatic disease. Chemokine receptors belong to the G-protein-coupled receptors (GPCRs) family that appear to be implicated in inflammatory diseases, tumor growth and metastasis. One of its members, C-C Chemokine receptor 7 (CCR7), binds chemokines CCL19 and CCL21, which are important for tissue homeostasis, immune surveillance and tumorigenesis. These receptors have been shown to induce the pathobiology of breast cancer due to their ability to induce cellular proliferation and migration upon the binding of the cognate chemokine receptors. The underlying signaling pathways and exact cellular interactions within this biological system are not fully understood and need further insights. Thus, in this review, we summarize the essential roles of CCR7 and its receptors in breast cancer progression. Furthermore, we discuss the mechanisms of regulation that may lead to novel opportunities for therapeutic intervention. Despite the enormous advances in our knowledge of the nature of the chemokines in breast cancer metastasis, research about the involvement of CCR7 in cancer progression is still limited. Therefore, further studies are essential to illustrate the distinct roles of CCR7 in cancer progression and validate its potential as a preventive bio-factor for human breast cancer metastasis by targeting chemokine receptor genes.

## 1. Introduction

Since their discovery over 30 years ago, numerous chemokines have been identified, with new roles emerging past the homing of leukocytes [1]. Chemokines or chemotactic cytokines constitute a large family of small (8–12 kDa) structurally related polypeptides that exert their functions by binding specific Gαi-protein-coupled chemokine receptors (GPCR) on the cell surface to induce chemotaxis [2]. Structurally, to date, more than 40 human chemokines have been described. The most widely used nomenclature subdivides chemokines into four subfamilies (CC, CXC, CX3C and XC) according to the position of the first two N-terminal cysteine residues in their amino acid sequence where C represents the cysteine and X is any amino acid [1].

Currently, 22 chemokine receptors have been identified in humans. These are G-protein-coupled receptors (GPCRs)-integral seven-transmembrane glycoproteins domains (connected by three intracellular and three extracellular loops), a short extracellular N-terminal that will bind to the ligand and an intracellular C-terminal coupled to the group of G-protein receptors. In the literature, four “atypical” receptors have been described capable of binding chemokines with high affinity with no signaling or signaling which is not mediated by G-proteins. This group of receptors has also been named “scavenger” receptors and recently renamed to “ACKR”, the acronym of atypical chemokine receptor [3]. Receptor–ligand interaction leads to signal transduction involving G-proteins which promotes the release of intracellular second messengers such as calcium, cyclic adenosine monophosphate (cAMP) and phosphoinositides (reviewed in [4]). Chemokines (chemotactic cytokines) are small heparin-binding proteins that are known to play a crucial role in directing the movement of cells throughout the body [5]. Most chemokines bind several receptors and a single receptor can often bind several chemokines, forming an intertwined web in which a sole role can be played by several elements. 

Chemokines and chemokine receptors are essential in dendritic cell (DC) maturation and (B and T) cells’ development [6,7]. Indeed, recent studies indicated that chemokines have a role in the chemotaxis of particular T cell or monocyte subsets that had not been identified previously [8,9]. The chemokine family can be either pro-inflammatory or homeostatic. The former is released due to a pathogen; an infection or other pro-inflammatory stimuli that causes the induction of chemokines that will direct the recruitment of leukocytes towards the site of injury [10]. Depending on the type of inflammation, a different immune cell subset will be recruited to the site [11], whilst the latter is constitutively expressed in specific tissues and has roles in tissue development (such as angiogenesis or neovascularization) or basal leukocyte migration. 

A considerable body of evidence highlights the importance of chemokines and their receptors during tumor progression and metastasis. Initially, chemokines selectively regulate the recruitment and trafficking of leukocyte subsets during homeostasis and inflammation [12,13]. However, it is becoming increasingly clear that they are also responsible for controlling the function of several tumor-promoting processes including host immune responses against malignant cells, cancer cell growth, angiogenesis and metastasis [14,15]. Chemokine receptors are further involved in the regulation of cancer progression through the recruitment of immune cell subsets. Importantly, there is a paucity of information from in vivo experimental systems regarding the specific function of individual chemokine receptors in cancer progression, whereas only very little data exist regarding their role in primary tumor formation.

The discovery that cancer cells overexpress C-C Chemokine receptor 7 (CCR7), which directs them to organs that express their ligands CCL21 and CCL19, led to an increase in reports confirming that chemokine receptors were present in a non-random manner in many other cancers. A positive correlation between chemokine receptor expression and worse prognosis has been found in most but not all cancers. In breast cancer, it has been proved that the chemokine receptors CXCR4, CCR7, CCR6 and CXCR3 and their ligands have been associated with metastasis [16,17]. Despite all the evidence linking CCR7 expression to a poorer prognosis, still much remains unknown about the exact mechanisms behind its upregulation and its role in breast cancer progression, which will be discussed in details in this review.

## 2. Chemokine Receptors in Cancer

The recruitment of immune cells to the tumor site is mediated by the actions of chemokines and their specific ligands, which have also been widely linked to cancer progression and metastasis. Upon the binding of the chemokine ligands, the receptor undergoes a conformational change. This results in the expression of several genes and activates a signaling cascade that, depending on the context, can stimulate cellular growth, migration, pseudopodia formation, adhesion, as well as angiostasis [18].

It was thought that metastasis was regulated through factors such as the size of the vessels or the difference in pressure between the blood and the organs [19]. However, because of the involvement of chemokine receptors in the migration of cells, they have been widely implicated in the metastasis of cancer cells throughout the body [10]. Tumor cells, through the expression of lymphoid chemokine receptors, may exploit the physiological lymphatic trafficking system to mediate invasion into the lymphatic vasculature [20]. Chemokine receptors can potentially facilitate tumor dissemination at numerous stages of the metastatic cascade, including vascular extravasation, protection from the host, adherence of tumor cells to endothelium, proliferation, colonization and angiogenesis [20,21]. To date, the most recognized receptor/ligand pairs in these phenomena include CXCR4/CXCL12 and CCR7/CCL21.

## 3. C-C Chemokine Receptor 7 (CCR7) in Cancer

C-C Chemokine receptor 7 (CCR7), is a GPCR commonly expressed by T-cell subset central memory cells, thymic T-cells, B cells, mature DCs and other rare cell subsets such as CD4+ CD25+ splenocytes [22,23,24,25]. 

CCL21 and CCL19 are constitutively expressed at the beginning of the lymphatic vessels and in lymphoid organs. They are present on several stromal cells and on the high endothelial venules (HEVs). Unlike CCL19, CCL21 binds to glycosaminoglycans (GAGs) and immobilizes on the surface of endothelial cells [26]. This means that only CCL21 is required for the intravasation into different lymphatic vessels [27]. Interestingly, the literature showed that the stimulation of CCR7 with both CCL19 and CCL21 ligands induces G-protein activation, ERK 1/2 signaling pathway, calcium mobilization and cell migration [28]. CCR7 desensitization and its ERK activation are mediated through β-arrestin, which may explain why the phosphorylation is four times stronger with CCL21, implying that CCL19 effects may be more transient than with CCL21 [29]. Internalized CCR7 is recycled back to transfected cell lines or the plasma membrane of primary T cells to participate in cell migration, whereas CCL19 is sorted to lysosomes for degradation [30].

While the overexpression of CXCR4/CXCL12 is correlated with the homing of cancer cells to the liver, lung, bone marrow and lymph nodes, the overexpression of the CCR7/CCL21 axis has mainly been related to lymph node metastasis [18]. It also plays a critical role in the progression of many different malignancies such as breast [31], gastric [32], skin (melanoma) [33], head and neck [34], lung [35], esophageal [36], hepatocellular [37], cervical [38], thyroid [39], tonsillar [40], colorectal [41] and prostate cancers [42] as summarized in (Table 1). In the majority of these cancers, larger tumor size and deeper invasion was associated with CCR7 expression [43]. Taken together, the CCR7-mediated cell migration underlies a broad range of immune system activities and therefore it is important to understand its mechanism.

### 3.1. Mode of Action of C-C Chemokine Receptor 7 (CCR7) in Migration and Adhesion

Cellular migration in vivo and in vitro is dependent on both the physical and biochemical properties of cells and the extracellular matrix (ECM) [65]. For cells to extravasate from the bloodstream and adhere to the endothelium, chemokines need to be bound to the GAGs present in the ECM [66]. This is an electrostatic interaction where the chemokine C-terminal region is positively charged due to the presence of basic amino acids such as lysine and arginine, whilst GAGs are highly negatively charged due to the presence of carboxylate and sulphate residues [66]. Recent work has shown that cells can sense the mechanical stiffness of their physical environment, and environmental stiffness can lead to changes in cellular gene expression and phenotype [67]. Moreover, chemokines can induce migration towards an increasing concentration of a chemoattractant or migration towards a decreasing concentration of a chemorepellent creating a concentration gradient called haptotaxis [68]. In mature DCs, for instance, immobilized CCL21 causes haptotaxis and integrin activation, while soluble CCL21 or CCL19 induce chemotaxis, and both can occur in combination [69]. 

The homing and migration of lymphocytes as well as CCR7-positive malignant cells metastasis into secondary lymphoid tissue (SLT) are precisely regulated by complex chemokine–chemokine receptor interaction in the microenvironment. CCR7-mediated T cell migration within SLT is critical for T cell activation and differentiation in adaptive immunity. Investigating the migratory response of CCR7 expressing T cells within a certain chemokine environment will lead to a better understanding of the mechanism of directional migration of T lymphocytes [70]. An investigation of CCR7 function in chemotaxing cells may also help to understand its role in cancer metastasis [71].

CCR7 is of particular interest in understanding metastasis because CD4+ T cells and DCs require CCR7 expression to migrate through lymphatic vessels [72]. The lymphatic vessels function as the sink for the interstitial flow; it has been postulated that interstitial flow and CCL21 act in conjunction to guide migrating tumor cells to lymphatics in the formation of metastases [73].

Pre-clinical studies have demonstrated that CCL19/CCL21 producing lymphatic endothelial cells can actively guide the chemotactic migration of CCR7-expressing tumor cells [33]. Additionally, CCL21 has been shown to promote the generation of new lymphoid-like structures within the tumor microenvironment, which are characterized by the infiltration of immune-suppressive T-regulatory cells and myeloid-derived suppressor cells [74]. However, the role of CCL21 during tumor progression remains somewhat controversial, with tumor-suppressive properties also been reported. CCL21 is a potent chemoattractant for tumor-infiltrating lymphocytes, which can exert a robust anti-tumor immune response especially during the early stages of tumor progression [75]. A recent clinical study reported an improved outcome associated with the increased infiltration of CCR7-positive T-cells within advanced colorectal carcinoma [76]. Within gastric cancers, CCR7 expression in the primary tumor was reported as the most important factor in determining lymph node metastasis. There is also preclinical evidence that CCR7 expression is a rate-limiting step for mediating the lymphatic spread of pancreatic ductal adenocarcinoma [77].

### 3.2. C-C Chemokine Receptor 7 (CCR7) and Angiogenesis

CCR7 has also been linked to the creation of new blood and lymphatic vessels in breast cancer patient samples, though the mechanism is still not well understood [44]. This lymphangiogenesis is mediated through VEGF-C and its receptor VEGFR-3 [44]. Indeed, the overexpression of this growth factor has been well documented to increase lymph node metastasis [78]. Interestingly, there are also studies suggesting that whenever tumor cells express CCL21 it has an anti-tumorigenic effect through the inhibition of angiogenesis and increase of leukocyte recruitment, in particular of CD8 + T-cells and DCs [79]. However, this immune response is suboptimal (as stated previously) and this could be due to the lack of maturation of the DCs.

## 4. C-C Chemokine Receptor 7 (CCR7) and Breast Cancer

The receptor CXCR4 is the most studied of all chemokine receptors in cancers. Its roles in breast cancer, including cell survival, proliferation, motility, invasion, angiogenesis, recruitment and activation of a number of different cell types, as well as metastasis are well documented [80]. CCR7 is often up-regulated together with CXCR4 in cancer [81]. In addition, these two receptors form a heterodimer on metastatic breast cancer cells, which activates alternative signaling pathways and promotes a metastatic phenotype [82]. 

In breast cancer, hypoxia has been shown to increase CCR7 expression through the hypoxia-inducible factor 1 (HIF-1)-mediated activation of the endothelin receptor A [83]. Epigenetic factors could also play a role in CCR7 upregulation, with histone deacetylation and DNA methylation playing a role in gene activation [84]. Despite all the evidence linking CCR7 expression to a poorer prognosis, still much remains unknown about the mechanisms behind its upregulation.

**Figure 1:**
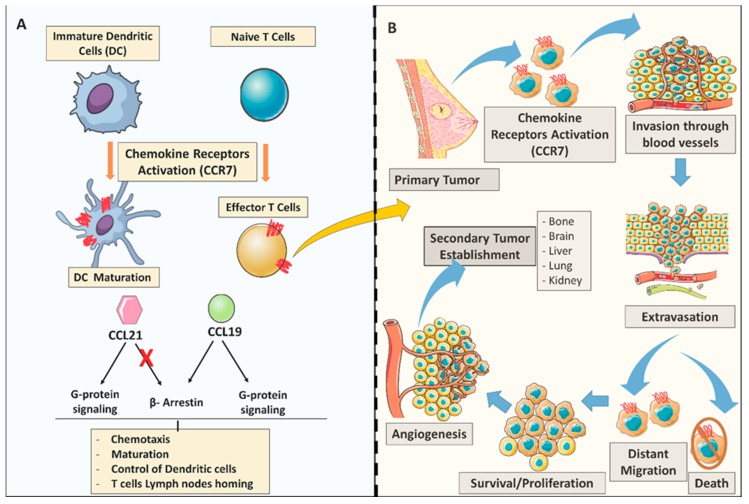
General C-C Chemokine receptor 7 (CCR7) chemokine dual role in the immune system and breast tumor metastasis. (**A**) CCR7 role in the adaptive immune system; CCL19/CCL21 chemokine expression activates CCR7 to promote dendritic cells (DC) and T-cell maturation. CCL21 and CCL19 chemokines determine differential signaling. Both chemokines CCL21 and CCL19 have the ability to display G-protein signaling cascades, however only CCL19 can trigger the β-arrestin recruitment. (**B**) Chemokine receptors in the metastatic process. (As illustrated) CCR7 chemokine receptor activation may play key roles in several steps during the process of metastasis; arrest; dissemination; invasion, extravasation; survival/proliferation) all under the control of the immune system trafficking to the site of inflammation.

The interaction between CCR7 and CCL21 has been shown to improve the immunogenicity of CCR7-expressing breast cancer cells [85]. It also induces actin polymerization and pseudopodia formation, resulting in increased cell motility as illustrated in (Figure 1) [86]. 

### 4.1. Molecular Aspects of C-C Chemokine Receptor 7 (CCR7) Signaling Cascades in Breast Cancer

Chemokine signaling in which a cell responds to a self-secreted chemokine is known as autocrine signaling, as opposed to paracrine signaling in which a cell responds to a chemokine secreted by a neighboring cell [87]. Both autocrine and paracrine signaling are well established mechanisms that drive tumor cell phenotype and migration.

There are a large number of chemokines that regulate the downstream effector molecules which may account for the various effects of these recipients in tumor pathology. To date, the effector molecules and unique mechanisms controlling cell chemotaxis, proliferation, survival and motility have been highly considered in chemokine receptor signaling in cancer [88,89].

Measuring chemokine ligands’ ability to induce early physiological changes, chemotaxis, actin polymerization and calcium mobilization is routinely performed in vitro to assess chemokine receptor functionality [90]. These findings provide evidence that CCR7 activation is cell-dependent via distinct mechanisms of actions. A number of key metastasis steps have been shown to facilitate tumor dissemination, including the endothelium adhesion of tumor cells, blood vessel extravasation, protection from host responses, angiogenesis, proliferation and metastatic colonization [91]. 

It is thought that chemokine responses are activated through key survival pathways such as the PI-3K and AKT, JAK/STAT, ERK 1/2, JNK, GSK-3α/β and MAP kinase [73,92,93]. Moreover, the exact signaling mediators in primary and invasive tumors are yet to be elucidated, although chemokine signals have been characterized in leukocytes with few currently available evidences on other cell types. The CCL21 binding to CCR7 has been reported to stimulate the activation phosphorylation of AKT (PI-3K pathway) that mediates a wide variety of intracellular targets to induce cell survival and inhibit apoptosis in many different types of cancer cells [94,95], as well as an implied effect on cell proliferation and motility [96]. This was further confirmed by the ability of CCR7 ligands to induce early migratory responses such as cytoskeleton remodeling and chemotaxis in metastatic breast cancer cells [97]. CCR5 and CCR7 enhance protooncogene c-Fos expression by JAK/STAT pathway in leukemia cells [98]. The activation of ERK in lymphoma and non-small lung cancer cells is also implicated in CCR3, CCR7 and CCR8 by inducing phosphorylation, proapoptotic protein inactivation and the activation of survival mechanisms [47,99]. 

Chemotactic responses and actin polymerization are not induced following CCR7 ligand-binding activation in non-metastatic breast cancer cells. Chemokine signaling pathways are mediated by the mobilization of the calcium flux through the activation of GPCRs, specifically the Gαi subunit that results from the inhibiting of adenylyl cyclase-mediated cAMP [100,101]. The pre-treatment with the Gαi inhibitor “pertussis toxin” impairs this calcium induction responsiveness. In addition, Adenylyl cyclase-mediated cAMP, stimulated by forskolin, is inhibited by CCL19 ligand treatment only in invasive breast cancer cells [102]. Therefore, functional Gαi signaling after chemokine treatment was only observed in the metastatic breast cancer cells. However, even after the addition of a phosphatidic acid, non-chemokine GPCR and non-metastatic cells did not respond. This might have been caused by the disruption of the upstream signaling events.

Hematopoietic and non-hematopoietic tumors metastatic to lymph nodes are correlated with tumor-dependent CCR7 expression. Physiologically, this indicates mature DCs and naive T lymphocytes recruitment to lymph nodes [73,103]. However, the induced expression of CCR7 in breast cancer cells switches metastasis from the lung to lymph nodes, indicating that the organ specificities of metastatic cancer cells might be sufficient by a single chemical receptor [31]. In some experimental settings, epithelial-to-mesenchymal transition (EMT) was triggered by a PI3K-mediated activation of snail and glycogen synthase kinase (GSk)-3β in hepatocellular carcinoma cells through CCL21/CCR7 and CXCL5/CXCR2 binding [104]. Moreover, TGF-β is reported to stimulate EMT through crosstalk with NF-κB signaling pathway in gastric cancer cells [51].

**Figure 2:**
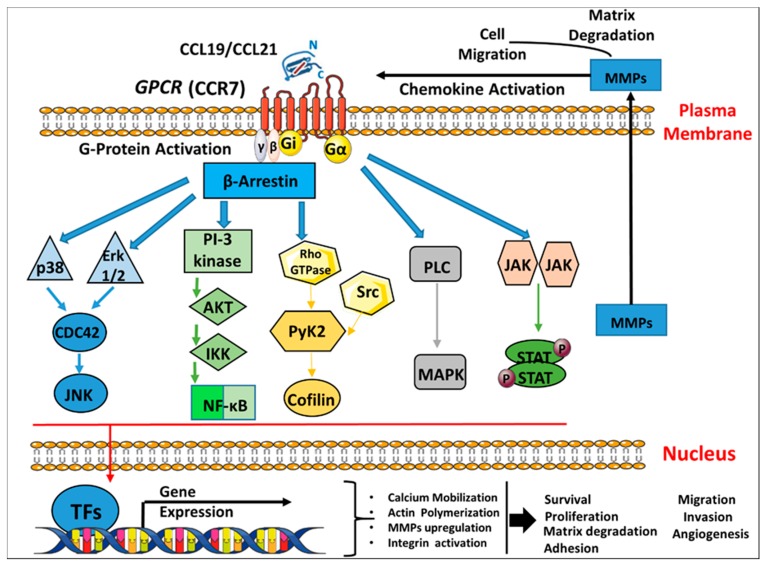
CCR7 chemokine receptor binding to the cognate ligands CCL19/CCL21 inducing signaling transduction pathways in breast cancer. Schematic representation of the seven transmembrane G-protein coupled receptor (CCR7) activation and internalization by ligand binding which triggers several signaling pathways cascades as follows: a. The binding of chemokine ligands CCL19/CCL21 to their CCR7 GPCRs leads to the activation of a G-α subunit and a Gi-βγ heterodimer. b. This initiates downstream signaling effectors that further lead to signaling cascades, such as the activation of ERK1/2, PI3K/AKT, Rho GTPases, MAPK, and JAK/STAT, which can promote the transcription and expression of different genes such as MMPs. c. All the above induce broad biological effects including chemotaxis, cell survival/proliferation, matrix degradation, reorganization of the actin cytoskeleton, adhesion, cell migration, invasion and angiogenesis. GPCR, G-protein-coupled receptors; ERK, extracellular signal-regulated kinase; JNK: c-Jun N-terminal kinase; PI3K, Phosphatidyl inositide-3-kinase; AKT, protein kinase B; NF-κB, nuclear factor-κB; Rho, Ras homolog gene family; PLC, phospholipase C; MAPK, mitogen-activated proteins kinase; JAK, Janus-activated kinase; TFs: Transcription factors, MMP, matrix metalloproteinase.

The pathways used by CCR7 to facilitate metastasis are not fully understood. It has been reported that CCR7 facilitates cancer progression by manipulating cancer cells’ anoikis and mobility [82] as well as controlling the tumor microenvironment, including the inflammatory responses, immune tolerance and T cell activation [105,106]. Downstream potential signaling mediators of CCR7 have been reported to be associated with metastasis, such as ALDH3A2, AnXA6, EpCAM, and PVR [107]. MMPs play an important role in tumor cell invasion and migration. It has been illustrated that CCR7 triggers AKT which eventually leads to the secretion of MMP-2/9. The silencing of CCR7 thus acts as a targeted therapy to inhibit AKT and MMPs expression and attenuates MCF-7 cell proliferation, invasion and EMT [45]. A schematic illustration of CCR7 binding to the cognate ligands CCL19/CCL21 to induce several signaling transduction pathways in breast cancer is shown in (Figure 2). 

CCR7 expression is regulated by transcription factors, epigenetic and miRNAs mechanisms. Transcription factors such as SP-1, AP-1, NF-κB and NF-AT1 can bind and regulate CCR7’ expression [108,109]. Nevertheless, it has not been well described whether these factors are correlated with tumor metastasis. The constituent expression of Ets-1, an oncogene that is involved in EMT, is well associated with the expression of CCR7 and cell migration activity in MDA-MB-231 [110,111].

Studies in the field of breast cancer have shown that the correlation between the expression of CCR7 and clinical pathogenicity in human breast cancer is important, including vascular invasion, lymph node metastasis and tumor grade [112]. However, other reports found contradictory results [113]. Hence, to gain greater certainty, more studies are recommended to determine the association between patient survival, recurrence risk and metastasis with the expression of CCR7. Moreover, CCR7 expression may enhance breast cancer cells’ migration to lymph nodes. Nevertheless, it is still not fully understood how this migration has contributed to a better prognosis. One explanation could be that positive CCR7 cancer cells primarily target metastasis to lymph nodes as well as empower the immune system to eliminate escaped cancerous cells [114]. Remarkably, Gracio et al. reported that the alternative splicing of CCR7 has been correlated to clinical results [115]. Therefore, the interactions between this essential post-transcriptional regulation and patient survival should be carefully investigated in correlation with CCR7 alternative splicing in breast cancer [116]. Nevertheless, this issue should be addressed in future studies to reveal the impact of CCR7 expression.

### 4.2. The Expression and Functional Role of C-C Chemokine Receptor 7 (CCR7) in Breast Cancer Cells in Vitro

The up-regulation of specific chemokine/receptor pairs has been reported in many human cancers including lung, breast, colon, prostate, gastric and melanoma [91,117], although at this point their precise mechanisms of action at each recurring event of metastasis are still being investigated. Recently, the expression of CXCR4 and CCR7 have received considerable attention in tumor cells for their direct involvement in metastasis. A variety of cancer cell lines and primary tumors tissues reported an expression of these chemical receptors and the interaction with the associated ligands to promote *the in vitro* and in vivo movement of cancer cells [38].

The CCR7 expression was evaluated in breast cancer cells in vitro to ascertain whether the rates of expression of a chemokine receptor contribute to the pathogenicity of breast cancer. A group of cell lines was used; each was originally derived from patients with different types of metastatic breast disease displaying various degrees of invasiveness. The examination of the CCR7 expression revealed significant levels of expression in different breast cell lines. Studies reported low CCR7 mRNA levels in MCF-7, MDA-MB-231 and T47D [18,118], no expression at mRNA levels in MDA-MB-231 [119], and inducible levels in MCF-7, MDA-MB-231, and SKBR3 [83]. Clinical studies revealed that breast cancer tumors show cytoplasmic CCR7 expression [120,121]. The same cytoplasmic-confined phenomena were also reported later in 4T1 cells [122]. These data confirm the internalization and activation of the CCR7 receptor upon ligand binding. It is also worth mentioning that CCR7 expression was detected with similar levels in both immortalized normal breast epithelial cells (MCF10A) as well as in a highly invasive breast cancer cell line (MDA-MB-231) [106]. These data strongly suggest that CCR7 expressions are not strictly limited to invasive cells and would not be a useful predictor of metastasis in aggressive breast cancer. Although the CCR7 physiological function is known in normal breast epithelial cells, their exact role remains unknown but valuable to be determined in non-metastatic breast cancer cells. Moreover, it can be predicted that cells gain selective advantages to proliferate, colonize, survive and migrate at secondary sites when chemokine receptors are activated through cancer progression.

### 4.3. The Role of C-C Chemokine Receptor 7 (CCR7) on Breast Cancer Metastasis in Vivo

Metastasis is one of the problematic tumor processes to study in vitro since the tumor progression relies on sequential events that are dependent on the characteristics of the tumor cells and their interactions with the tissue environment [123]. Therefore, more relevant studies of metastasis are thought to originate from those performed in vivo. Significantly, mice models of different human cancers have become a central part of many types of biomedical research as they provide the most experimentally accessible mammalian model that shares genes, physiology and organ structure with the human system [124].

To provide further insight into chemokine-mediated metastatic mechanisms, the expression of CCR7 on mammary epithelial cell lines was modulated using an shRNA system based on the ability to deliver and stably express shRNAs, to study the effect of chemokine modulation on the metastatic propensity of breast cancer cells in vivo [125]. The silencing of CCR7 decreased the in vitro proliferation, migration and invasion of CCL21/CCL19-induced MCF-7 and MDA-MB-231 breast cancer cells [45,126]. Furthermore, the depletion of CCR7 in BALB/c mice inhibited orthotopically injected 4T1 cells metastasis, while tail veins’ injection had no impact on breast cancer metastasis. These outcomes were consistent with the previous findings, showing that CCR7 did not affect liver and lung metastasis when pancreatic cancer cells (TD-2) were injected via tail veins, but rather increased B16 cell metastasis to lymph nodes [127]. On the other hand, CCR7 overexpression in MMTV-PyMT-mediated breast cancer cells, injected orthotopically into FVB mice, significantly increased lymph node metastasis [31]. 

CCR7 expression has been reported in particular to increase B16 metastatic capacity via the lymphatic system but not through a blood-borne pathway [128]. At first, CCR7 had a significant role as stimulated DCs migrated through various lymph vessels to specific lymph nodes. Subsequently, it was suggested that neoplastic cells also enter lymph vessels to improve lymphatic dissemination, shown for B16 melanoma cells [129]. The formation of the CCR7 metastatic lymph node expressing B16 cells was effectively blocked by the neutralization of anti-CCL21 [129], which confirmed the role of CCR7 during metastasis. Given the contrasting studies, the exact relationship between the expression of chemokine in metastatic tumor progression and their potential role still requires considerable attention. 

Higher levels of CCR7 mRNA was detected in cancer tissue compared to the normal counterpart lung tissues, suggesting that CCR7 expression could be used to predict the metastasis of the lymph node before surgery as a diagnostic tool to improve the overall treatment plan for non-small cell lung cancer (NSCLC) [55]. Moreover, the CCR7 presence was directly related to the lymph node metastases development in NSCLC [35]. In addition, the expression of chemokine receptors in clinical samples may help correlate their levels with the outcome of cancer patients. Interestingly, based on the expression status of hormonal receptors, triple-negative breast cancer (TNBC) is associated with poor prognosis, higher metastasis and worse outcomes than other subtypes due to the absence of efficient targeted therapies [130]. In breast cancer tissues, CCR7 has been highly expressed especially in the luminal B-type and TNBC compared to the luminal A type [114,131]. Therefore, tumor microenvironment signals are likely to be interpreted differently in luminal-B cells than those in luminal-A cells. Thus, the tumor microenvironment factors do not inhibit the responsiveness of luminal-B cells to chemotactic cues mediated through the CCR7/CCL21 axis [46].

Several experimental approaches using specific chemokine antagonists, neutralizing antibodies, knockout mice and targeted gene disruptions have been reported to be useful tools for discerning the role of chemokines and their receptors in vivo [132,133]. Therefore, these methods may potentially provide a basis for the development of future therapeutic agents. Studies involving the inhibition of CCR7 expression on cancer cells are yet to be explored at both the in vitro and in vivo level.

## 5. Therapy and Future Directions

For most chemokine receptors, removing their cytoplasmic tail impairs endocytosis [134,135]. For CCR7, the deletion of the entire cytoplasmic tail did not totally block the receptor endocytosis and recycling but also eliminated the CCL19/CCL21-induced chemotaxis. Indeed, in vivo CCR7 C-terminal partial truncation decreases the receptor internalization, while maintaining the chemotaxis ability to CCR7 ligands [135], and prevents lymphocyte adhesion and extravasation to the HEVs [136]. Additional studies reported that the intra-tumoral injection of recombinant CCL21 significantly delayed tumor progression and stimulated cytotoxic immune responses [85,137]. Moreover, in vitro experiments have demonstrated that blocking the CCR7 receptor decreases the incidence of metastasis [86,121].

Initial reports suggested that CCL21 and CCL19 were anti-tumorigenic, as they could attract lymphocytes that would attack the tumor [79]. However, it has been described that in CCL21-expressing tumors, the chemokines create a tolerogenic environment where the lymphocytes behave similarly to the lymph node stroma [74]. Preliminary studies into the use of CSC vaccines by pulsing DCs with CSC lysates (based on the expression of aldehyde dehydrogenase) to generate B cell-mediated immunity against CSCs in mouse models of melanoma and squamous cell carcinoma have been conducted. The application of the DC vaccine reduced the development of primary tumors and metastasis and interestingly, this was associated with a significant decrease in the expression of CCR7 and its ligand CCL21 [138]. Therefore, the inhibition of CCR7 may potentially be used in combination with conventional chemotherapeutics in a multipronged approach to eliminate both rapidly dividing bulk tumor cells and quiescent stem-like cells.

The function of CCR7/CCL21 in cancer appears to be controversial; it may play a pro- or anti-tumor function depending on where it is expressed. In tumor cells, it induces development and progression, whereas when expressed by immune cells such as dendritic or T cells, anti-tumor responses are detected [91]. Moreover, the response of the host to recognize poorly immunogenic tumors is one of the challenges in developing cancer immunotherapy. DCs are bone-marrow-generated leukocytes with high levels of MHC expression and co-stimulatory molecules [139]. Their ability to consume, process and present antigens, combined with their ability to activate and expand antigen-specific T cells, make them a promising target for the development of cancer immunotherapy strategies [140]. Recent studies in cancer immunotherapy have investigated the use of WT CCL21 as a chemoattractant of dendritic and T cells to the tumor [141]. For example, the FDA has approved immunotherapy using DCs against metastatic, asymptomatic prostate cancer [142]. The vaccine was considered safe and showed an encouraging response to the tumor although these trials are still underway [143,144]. A more advanced delivery system using immunocompetent mice was used to evaluate this either by encoding CCL21 in an adeno-recombinant associated virus to enable its transduction following the intra-tumoral implant [145], or only by directly injecting CCL21 into the tumor cells, both resulting in reduced tumor development [146]. Another alternative is to inject exaggerated CCL21-transduced DCs or fibroblasts that mediate the functional differentiation and recruitment of innate and adaptive immune cells into the tumor, which has been shown to destroy cancer cells [147]. In summary, the dual roles of the CCR7/CCL21 axis in protective immunity and tumor promotion suggest that their targeted therapies must be carefully evaluated.

This comprehensive review provided an insight into chemokine-mediated mechanisms performed in breast cancer cells both in vitro and in vivo, specifically CCR7; there are still important questions that remain to be answered. It will be valuable to gain a broader understanding of the functional role of chemokines and their receptors in different cancers other than breast cancer. In addition, given the complex process of metastasis, it is likely that chemokine receptors may function at several steps and therefore, the use of specific in vivo models may assist in elucidating the precise stage at which chemokines and their receptors influence the metastatic pathway. Finally, it would be recommended to examine the effect of chemokine receptor expression on the growth of primary tumors using an orthotopic cancer model to verify the precise role of chemokines during metastasis. The understanding of chemokine-mediated pathways and consequently, their potential disruption may provide useful information for the design of new strategies to treat breast cancer.

## 6. Conclusions

In recent decades, there have been vast advances in our knowledge regarding the roles of the chemokines in cancer progression. In addition, most chemokines have been reported to act as promoting factors for tumor growth and proliferation, angiogenesis and metastasis. This could be due to the promiscuity of chemokine and chemokine receptor interactions in cancer cells. 

Participation in cancer cell invasion, metastases and tumor development was reported with the CC Chemokine receptor 7 (CCR7). CCR7 and its receptors have been particularly reported to stimulate the tumorigenesis of breast cancer. It also played a novel role in inducing lymphangiogenesis in the experimental model of breast cancer. It was also accompanied with EMT in human breast carcinoma. Furthermore, CCR7 has been involved in the metastasis of the lymph nodes, invasion, migration and EMT. Findings showed that the EMT progression in breast cancer cells was controlled by the CCL19/CCR7 axis and facilitated the invasion and migration process of tumor cells by triggering several signaling pathways. On the basis of this review, CCR7 has a critical role in breast cancer progression. Thus, it might be elucidating that CCR7 could be a novel target for tumor therapy when used in combination with chemotherapy or immune checkpoint therapy. For verification of CCR7 and its ligands as a therapeutic target for breast cancer progression, further studies are required to illustrate their function and the mechanism of their actions involved in breast cancer progression.

## Figures and Tables

**Table 1:** The roles of C-C Chemokine receptor 7 (CCR7)/CCL21 in various human cancers.

Tumor Type	Observations	References
Breast Cancer	Correlates and promotes lymphogenesis and metastasis. CCL21 induces actin polymerization and migration.	[18,44,45,46]
Human Bladder Cancer	Enhances the migration, invasion and tumor proliferation/ correlates with poor prognosis.	[43,47]
Cervical Cancer	Metastasis and poor prognosis.	[38,40]
Colorectal Cancer	Poor prognosis and metastasis.	[48,49,50]
Gastric Cancer	Overall poor survival as well as metastasis and EMT.	[51,52]
Head and Neck Cell Carcinoma	CCR7/CCL21 correlates with metastasis.	[53,54]
Lung Cancer	Tumor dissemination and metastasis.	[55,56]
Lymphomas	Poor prognosis and tumor dissemination.	[57,58]
Melanomas	Metastasis and poor outcome.	[18,59]
Esophageal Cancer	Expression of CCR7 correlates with poor prognosis and metastasis. CCL21 induces pseudopodia formation, cell metastasis and angiogenesis.	[60,61,62]
Prostate Cancer	Tumor growth, metastasis and poor survival through lymphatic metastasis.	[63,64]
